# 

**Published:** 1899-09

**Authors:** 


### CONTENTS:

### SELECTIONS.

Article I.—National Association of Dental Faculties .......... 193
" II.—Pressure Annesthesia. Dr. Wm. J. Morton .......... 209
" III.—Practical Results of Higher Education .......... 217
" IV.—Is Dentistry a Remunerative Profession? .......... 222
" V.—Eailures in Bridge-Work. Dr. F. M. Willis .......... 226
" VI.—Alveolar Hemorrhage. Dr. W. A. White .......... 229
" VII.—Combination Filling. F. B. Spooner, D. D. S .......... 231
" VIII.—A Peculiar Restoration. Ellison Hillyer, D. D. S. .......... 234
" IX.—Dentistry in the Army. Homer C. Croscup, D. D. S. .......... 236

### MONTHLY SUMMARY.

Alveolar Hemorrhage .......... 239
To Apply the Rubber Dam .......... 240
Arsenical Poisoning Cured by Orthoform .......... 240

